# Pigs Ferment Enzymatically Digestible Starch when it Is Substituted for Resistant Starch

**DOI:** 10.1093/jn/nxz072

**Published:** 2019-06-04

**Authors:** Rik J J van Erp, Sonja de Vries, Theo A T G van Kempen, Walter J J Gerrits

**Affiliations:** 1 Trouw Nutrition, Amersfoort, The Netherlands; 2 Animal Nutrition Group, Wageningen University, Wageningen, The Netherlands; 3 North Carolina State University, Raleigh, NC

**Keywords:** feed intake, resistant starch, feeding behavior, nutrient digestibility, adaptation

## Abstract

**Background:**

Feeding behavior is controlled by satiety mechanisms, which are affected by the extent of starch digestion, and thus resistant starch (RS) intake. Alterations in feeding behavior to changes in RS intake may depend on the adaptation of processes involved when shifting from starch digestion to fermentation or vice versa.

**Objectives:**

The aim of this study was to investigate how growing pigs adapt their feeding behavior in response to increasing and decreasing dietary RS concentrations.

**Methods:**

Thirty-six groups of 6 pigs (25.4 ± 2.8 kg; Hypor Libra × Hypor Maxter; male:female, 1:1) were fed diets containing 50% high-amylose maize starch (high RS; HRS) or waxy maize starch (low RS; LRS). Over 28 d, diets were exchanged following a 5-step titration (25% per step) that was executed in the upward (LH) or downward direction (HL). Twelve groups received a control diet to correct for changes over time. Individual feeding behavior and total tract starch digestion and fermentation were evaluated. The response in each parameter to increasing dietary HRS inclusion was estimated through the use of linear regression procedures, and tested for titration direction and sex effects.

**Results:**

Complete substitution of LRS with HRS increased the proportion of starch fermented, which was greater in LH pigs than in HL pigs (17.6% compared with 8.18%; *P *< 0.001), and decreased the feed intake (106 g/d; *P *= 0.021) and meal size (12.6 g; *P *< 0.001) of LH pigs, but not of HL pigs. In LH pigs, the size of the starch fermentation response positively correlated with the size of the feed intake response (*r* = 0.90, *P *< 0.001).

**Conclusions:**

The attenuated response in starch fermentation in HL pigs indicates that pigs adapt more slowly to dietary supply of digestible starch than to RS, consequently resulting in fermentation of enzymatically digestible starch. Feed intake and feeding behavior only changed in pigs poorly adapting to RS, indicating that adequacy of adaptation, rather than RS itself, drives feed intake. These findings stress the importance of diet history for nutrient digestion and feeding behavior.

## Introduction

Increasing the dietary amount of resistant starch (RS) at the expense of digestible starch may reduce feeding motivation in pigs (1, 2), thereby affecting their feeding behavior. The literature on the effect of RS on satiety on humans is inconclusive, as some studies report an increase in satiety following RS consumption (3–5), whereas others reported little or no effect (6, 7). Substituting digestible starch with RS decreases postprandial glucose appearance, which reduces the release of satiety hormones [e.g., (5, 8–10)] stimulating the ileal brake mechanism (11). Consequently, meal size may increase, as satiation decreases. It is hypothesized, however, that the potential bulking properties of RS, similar to other fibrous feed sources (12), may increase satiation through gastric distension (13). However, the bulking capacity of RS when compared with typical high-bulk fiber sources, such as sugar beet pulp or wheat bran, is low (12), and therefore its volumetric effect on short-term feed intake may be limited. The presence of products from microbial fermentation in the distal gastrointestinal tract may also slow down gastric emptying (14, 15), and activate intestinal brake mechanisms (16–18), thereby decreasing digesta transit in the upper gastrointestinal tract and stimulating satiation. In addition, the prolonged energy supply to the body by SCFAs resulting from microbial fermentation of RS (10) may induce long-term postprandial satiety, increasing the interval between meals.

Prolonged exposure (i.e., 12 wk) of pigs to a high-RS diet compared with a low-RS diet increased meal size by 10% and decreased meal frequency by 2 meals/d in growing pigs, whereas daily feed intake was not affected (19). We hypothesize, however, that these alterations in feeding behavior to changes in RS intake are dynamic, depending on the adaptation processes involved when shifting from enzymatic digestion to microbial fermentation or vice versa. To gain understanding of the mode of action of the effects of RS on feeding behavior, we investigated the effects of changes in dietary RS intake on feeding behavior of growing pigs. As these effects may depend on the direction of adaptation, we tested effects of increasing and decreasing dietary RS concentration.

## Methods

The experimental protocol was approved by the Animal Care and Use Committee of Utrecht University, Utrecht, The Netherlands; the experiment was performed at the experimental facilities of Nutreco NV, Sint Anthonis, The Netherlands.

For this study, 288 pigs (Hypor Libra × Hypor Maxter) were selected at 9 wk of age, blocked in 3 weight categories—light (22.2 ± 1.1 kg), medium (25.3 ± 0.8 kg), and heavy (28.7 ± 1.3 kg)—and assigned to 48 pens (6 pigs/pen) in 1 of 6 departments where the animals were housed. Pens were 4.70 m × 2.40 m with 60% slatted floors and were equipped with an electronic single-space feeding station (EFS) for fattening pigs (Schauer). Sex was equally divided over pens (1:1). After an adaptation period of 10 d during which pigs were fed a commercial diet, 36 pens (18 pens each) were assigned to 1 of 2 diets (**Table 1**), containing either 50% high-amylose maize starch (high RS, HR S; Roquette) or 50% waxy maize starch (low RS, LRS; Roquette). The rate (*k*; LRS: 3.14%/min; HRS: 2.19%/min) and extent (*D*; LRS: 99.9%; HRS: 65.8%) of starch digestion of each source were analyzed through the use of an adapted in vitro procedure modified from Englyst et al. (20), and determined by fitting the following first-order kinetic model:

(1) \begin{equation*} {\rm{starch\ degraded\ }}\left( {{\rm{\% \ at\ time}}\ t} \right)\ = \ D \times \left( {1 - } \right.{e^{ - k*t}}) \end{equation*}

**TABLE 1:** Ingredient composition and analyzed chemical composition of experimental diets^1^

	Diets	
	HRS	LRS
Ingredient, g/kg		
Waxy maize starch^2^	500	—
High amylose maize starch^3^	—	497
Water^4^	25.5	28.5
Rape seed meal	130	130
Sunflower seed meal	130	130
Wheat gluten meal	100	100
Palm oil	33.0	33.0
Molasses, cane	25.0	25.0
Potato protein	15.0	15.0
Premix^5^	12.5	12.5
Monocalcium phosphate	7.2	7.2
Calcium carbonate	6.7	6.7
l-Lysine	6.0	6.0
Sodium bicarbonate	5.5	5.5
l-Threonine	0.9	0.9
dl-Methionine	0.2	0.2
l-Tryptophan	0.2	0.2
Choline chloride	0.1	0.1
Phytase	0.1	0.1
Titanium dioxide	2.0	2.0
Analyzed chemical composition, g/kg as fed		
DM	888	890
Crude fat	51.0	52.0
Crude protein	179	181
Neutral detergent fiber	102	111
Starch	444	456
Gross energy (MJ/kg as fed)	17.7	17.7

1 DM, dry matter; HRS, high resistant starch; LRS, low resistant starch.

2 Roquette Amido di Mais Waxu N-200.

3 Roquette Amido di Mais Amylo N-400.

4 Water was included to compensate for a lower DM content of high-amylose maize starch.

5 Supplied per kg of feed: citric acid, 111 mg; propyl gallate, 69 mg; butylhydroxytoluene, 151 mg; sepiolite, 158 g; retinyl acetate, 8000 IU; cholecalciferol, 1600 IU; all-rac-α-tocopheryl acetate, 7.5 IU; menadione nicotinamide bisulfite, 160 mg; thiamin mononitrate, 80 mg; riboflavin, 400 mg; calcium-d-pantothenate, 1.3 g; choline chloride, 12 g; niacinamide, 1.6 g; pyridoxine hydrochloride, 120 mg; folic acid, 120 mg; cyanocobalamin, 1.6 mg; biotin, 12 mg; betaine hydrochloride, 7.9 mg; iron(II) sulfate, 8 g; calcium iodate, 80 mg; copper(II) sulfate, 12 g; manganese(II) oxide, 2.4 g; zinc oxide, 8 g; sodium selenite, 24 mg;

Over a 28-d period, HRS and LRS were interchanged in 5 steps, either in an upwards (low to high; LH) or downwards (high to low; HL) direction. The first titration step lasted for 8 d. For the subsequent titration steps, a length of 5 d was considered sufficient, as the increment per titration step was small. Pigs in the remaining 12 pens received a 50/50 mixture of both diets as a control treatment until the end of the experiment to control for changes in feeding behavior and digestive processes over time. Animals were exposed to 16 h of light (from 0600 to 2200 h) and 8 h of darkness. Temperature was set at 23°C.

### Diets and feeding

Five diets differing in LRS:HRS ratios (100:0, 75:25, 50:50, 75:25, and 0:100) were formulated to meet or exceed nutrient requirements for growing pigs (21), and pelleted. Titanium dioxide (2 g/kg) was added as indigestible marker in all diets to determine apparent total tract digestibility. During the adaptation period, pigs were fed a commercial diet (crude protein 172 g/kg, net energy 9.17 MJ/kg; ABZ). Feed and water were available ad libitum throughout the experiment.

### Measurements

Pigs were weighed at the start and end of the experiment. Each pig received an electronic ear transponder corresponding to a unique identification number that was read by 2 antennas in the EFS. Data generated by the EFSs were continuously stored: the pig's identification number, the date, the time of entry and exit per visit, and amount of feed consumed per visit. On the last 2 d of each titration step, grab fecal samples were collected from the floor at 0700 h and 1500 h. All feces not visually contaminated with urine were collected to obtain a representative sample for all the animals in a pen. The remaining feces on the floor were removed each time after sample collection. During the other days, floors were cleaned twice a day, in the morning and evening. Samples were homogenized, pooled by pen per titration step, and stored at −20°C. Prior to analysis, they were freeze-dried, and ground to pass a 1 mm screen. For each weight group, fecal samples of 3 randomly selected pens from each titration direction (*n* = 9) and 2 randomly selected pens from the control group (*n* = 6) were subsequently analyzed. Diets and feces were analyzed for contents of dry matter (DM) (22), nitrogen and carbon (23), starch (24), and titanium (25). In starch sources, and in ball-milled nonstarch diet ingredients, complete diets, and freeze-dried feces, ^13^C enrichment was analyzed by combustion isotope ratio MS with the use of a Delta V Advantage isotope-ratio mass spectrometer (Thermo Scientific). All analyses were carried out in duplicate.

### Data screening and calculations

Boundaries set to clean the raw EFS data were based on visual plots of visit time × feed intake, and visit time × rate of feed intake (RFI). The registration of a visit at the EFS was considered incorrect if: visit time <60 s and feed intake >200 g; visit time >60 s and RFI >120 g/min; visit time >300 s and feed intake <40 g. Applying these criteria, 557 out of 461,750 observations were discarded. Feeder visits themselves were not considered as meals, because they can occur so close together that from a digestive perspective they should be considered as a single meal. Hence, when the interval between 2 successive visits did not exceed the meal criterion (320 s), visits were added together and counted as a single meal. Meal criteria were estimated individually on data obtained during the last 5 d of the pre-experimental period. Individual meal criteria were averaged to 1 meal criterion, to avoid confounding effects of individual meal criteria on meal parameter estimates. The meal criterion was estimated by fitting a model consisting of 2 Gaussian and 1 Weibull distribution (2) to the distribution of log-transformed intervals between 2 successive visits of each individual pig (26).

(2) \begin{eqnarray*}
{y} &=& p\, \left({{1/{\sigma_1}\sqrt {2\pi}} {\rm e}^{ -{{\left({x - {\mu _1}} \right)}^2}/2{\sigma _1}^2}}\right) + q \left( {1/{\sigma_1}\sqrt {2\pi}} {\rm e}^{ -{{\left({x - {\mu _2}} \right)}^2}/2{\sigma _2}^2}\right)\nonumber\\
&& +\, {\left( {1 - p - q }\right)} \left( {{\propto}/{\beta}^{\propto}}\right){x^{\propto} - {1 _e}^ - {x/{\beta}}^{\propto}}
\end{eqnarray*}

where *y* is the probability density of log (interval length) in seconds, *p, q*, and 1 – *p* – *q* are the proportions of intervals in each distribution, *x* is the log (interval length) in seconds, σ_1_ and σ_2_, and μ_1_ and μ_2_, are the respective SD and mean of the first and second distribution, and α and β are the respective scale and shape parameter of the third distribution (**Supplemental Figure 1**). The first curve describes the short, within-meal intervals; the second curve is suggested to be associated with drinking behavior; the third curve describes the long intermeal intervals (26). The intersection between the second and third curves was used to set the meal criterion (27). Meal size and duration were calculated as the respective sum of feed intake and visit duration within 1 meal. Intermeal interval was calculated as the average time between 2 successive meals. RFI was calculated as daily feed intake divided by total time spent eating. Average daily feed intake (ADFI), RFI, time spent eating, daily number of visits, and meal frequency, size, and duration were calculated per pig, per titration step. For both titration directions, only the last 2 d of each titration step were used for calculation of the mean value per titration step. For the control group all data were used for calculations, except for the first 6 d to allow pigs to adapt to the experimental diet.

Apparent total tract digestibility (ATTD) of nutrients were calculated from the following equation (28):

(3) \begin{eqnarray*}
&& {\rm{Nutrient\ dissappearance\ }}\left( {{\rm{\% \ of\ intake}}} \right)\nonumber\\
&&\quad = \left( {1 - \left( {\frac{{{\rm{Nutrien}}{{\rm{t}}_{{\rm{feces}}}}}}{{{\rm{T}}{{\rm{i}}_{{\rm{feces\ }}}}}}/\frac{{{\rm{Nutrien}}{{\rm{t}}_{{\rm{feed}}}}}}{{{\rm{T}}{{\rm{i}}_{{\rm{feed}}}}}}} \right)} \right)\ \times 100
\end{eqnarray*}

where Nutrient_feces_ is the nutrient concentration in the feces (g/kg DM), Ti_feces_ is the titanium concentration in the feces (g/kg DM), Nutrient_feed_ is the nutrient concentration in the feed (g/kg DM), and Ti_feed_ is the titanium concentration in the feed (g/kg DM).

Total tract starch fermentation was calculated following the method of Gerrits et al. (29). Briefly, diets were designed to have a contrast in natural ^13^C enrichment between the maize starch (waxy maize starch: 1.0932%; high amylose maize starch: 1.0934%) and the nonstarch ingredients of the diet (1.0767%). Consequently, an increase in fecal ^13^C enrichment can result from greater fecal excretion of ^13^C from dietary starch (degradation products), or by starch-derived ^13^C incorporated in microbial biomass. Total fecal starch, carbon content, and ^13^C enrichment were analyzed to calculate the amount of starch-derived carbon incorporated in microbial biomass. Finally, the amount of starch fermented was calculated by assuming that for 1 g of microbial biomass, 5 g of carbon are required (29); this assumption is based on the microbial efficiency of high-starch diets in dairy cows (30) or calculated (31) by assuming a fixed conversion of carbohydrates to microbial biomass (0.3 kJ fecal biomass/kJ carbohydrate) (32), and energy content of carbohydrates (15.56 kJ/g) (33) and biomass (23.13 kJ/g) (34).

For each pig, the responses in feed intake, digestibility, and fermentation parameters to decreasing or increasing dietary HRS concentrations were estimated by regression procedures performed with SAS version 9.4 for Windows (SAS Institute) following the model:

(4) \begin{equation*} y\ = \ a + \ \beta x \end{equation*}

where *y* = mean of response variable, *a* = intercept (0% inclusion of HRS), β = slope of regression line, i.e., change per percentage inclusion of HRS, and *x* = proportion of HRS (% of total starch) or time (d). For control pigs, a similar regression was performed, taking observations at the start of the experimental period as the intercept *a*, and time (d) as regressor *x*. The calculation of responses related to increasing dietary HRS concentrations and intercepts of each pig (μ) were corrected for significant time-related changes observed in the control pigs per titration step as follows; LH: μ – β_control_; HL: μ + β_control_. The results of the control group are expressed as daily changes in all parameters, and results of LH and HL groups are expressed as the change in size of each parameter for the complete substitution of LRS with HRS (100% HRS inclusion).

### Statistical analysis

All statistical analyses were performed with SAS version 9.4 for Windows. For all feeding behavior data, each animal was considered as an experimental unit. Response estimates for the control group (*n* = 72), and responses of LH and HL pigs (*n* = 108) corrected for time-related effects calculated for feeding behavior parameters were analyzed through the use of a general linear mixed model (5) to check if the response was significantly different from zero. Pen was modeled as random G-side effect to account for correlation among pigs within pen. To verify linearity, individual data were checked visually, and tested for quadratic effects by adding a quadratic term to the regression model, which was significant for <15% of the population for all parameters. The homogeneity and normality of model residuals were checked visually with the UNIVARIATE procedure.

(5) \begin{equation*} {Y_{ijklmn}} = \mu + {T_j} + {S_k} + {C_i} + {D_l} + {P_m} + {e_{ijklmn}} \end{equation*}

where *Y_ijklm_* is a dependent variable (response to 100% substitution of LRS with HRS), μ is the overall mean; *T_j_* (titration direction), *S_k_* (sex), and *C_i_* (body weight class) are fixed effects; and *D_l_* (department) and *P_m_* (pen) are random effects.

Data on digestibility and fermentation parameters were measured per pen (control: *n* = 6; HL: *n* = 9; LH: *n* = 9); therefore, the effect of sex and pen were removed from the model. Pearson correlation coefficients were estimated to investigate the relation between response parameters.

Intercepts are presented as mean ± pooled SEM. Response parameters (β) are reported as LS means ± pooled SEM, and expressed as either the increment in response parameter per day for the control pigs, or expressed as the change in the size of the parameter for the complete substitution of LRS with HRS. In this way, the responses bear the same sign, independent of titration direction, and are easier to interpret. The change in feed intake and starch fermentation of each titration direction over time (per titration step) are presented in **Supplemental Figures 2** and **3** to exemplify the results. Differences were considered significant if *P *< 0.05.

## Results

### General

Observations of 8 pigs were discarded; 6 pigs suffered from lameness (2 control pigs, 2 LH pigs, 2 HL pigs), and 2 pigs from a rectal prolapse (1 LH pig, 1 HL pig). One pig died before the start of the experimental period for unknown reasons (1 control pig). Body weight did not differ among treatments, and averaged (± = standard error) 25.4 ± 0.17 kg at the start and 61.0 ± 0.36 kg at the end of the experiment. Average daily gain during the experimental period of control pigs (957 ± 11.8 g/d), HL pigs (965 ± 11.1 g/d), and LH pigs (966 ± 12.0 g/d) did not differ. Weight class did not affect any of the response parameters.

### Digestibility parameters

#### Changes over time (control group)

Apparent total tract digestibility of DM and nitrogen of control pigs increased over the 28-d study period (DM: 0.08%-unit/d, *P *< 0.001; nitrogen: 0.19%-unit/d, *P *= 0.001; **Supplemental Table 1**). Total tract starch digestibility increased (0.01%-unit/d, *P *= 0.027), whereas starch fermentation decreased over time (0.12%-unit/d, *P *= 0.029).

#### Titration direction

**FIGURE 1:**
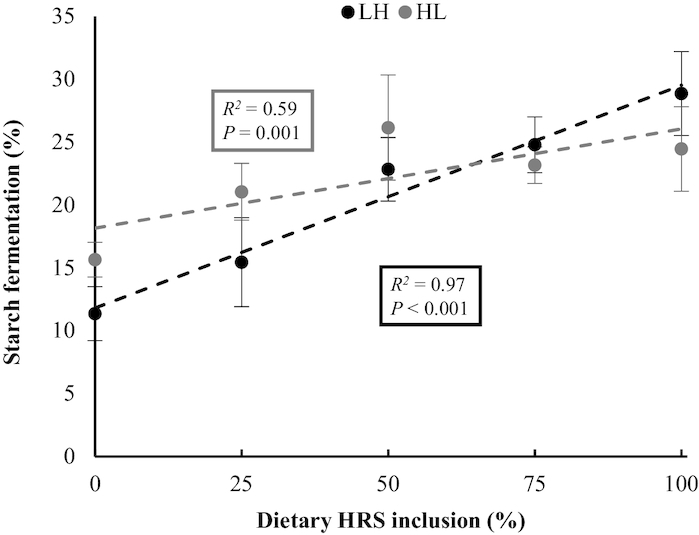
Response in dietary starch fermentation in growing pigs to incremental dietary concentrations of high amylose maize starch (HRS), when substituting waxy maize starch (LRS) with high amylose maize starch (low to high RS; LH) or vice versa (high to low RS; HL) in 5 steps from 0% to 100% over a period of 28 d. Dietary inclusion of starch sources was 50% (w/w, as fed) in all diets. Starch fermentation was calculated from the contrast in natural ^13^C enrichment between starch and nonstarch dietary components, by assuming 5 g starch fermented/g C from starch fermentation in feces (29). Data were corrected for the time-related effect on starch fermentation measured in control groups (*n* = 6) receiving a diet of 50% HRS and 50% LRS during the complete experiment. Data are presented as least square means ± pooled SEM, *n* = 9 replicates (6 pigs/replicate). HL, high RS to low RS titration; HRS, high resistant starch; LH, low RS to high RS titration; LRS, low resistant starch.

For both LH and HL pigs, complete substitution of LRS with HRS decreased the ATTD of DM, nitrogen, and starch (*P < *0.010), and increased total tract starch fermentation (*P* < 0.001) (**Table 2**; **Figure 1**). Observed responses in ATTD of DM, nitrogen, and total tract starch fermentation to complete substitution of LRS with HRS were larger in LH pigs than in HL pigs (*P < *0.010), whereas the observed response in ATTD of starch digestion was smaller in LH pigs than in HL pigs (*P *= 0.002).

**TABLE 2:** Intercepts and responses in nutrient disappearance and feeding behavior parameters in growing pigs (25–60 kg) to incremental dietary concentrations of high amylose maize (HRS), when substituting waxy maize starch (LRS) with high amylose maize starch (LH) or vice versa (HL) in 5 steps from 0% to 100% over a period of 28 d^1^

				Response (0–100% HRS)^2^				
	Intercept (0% HRS)						*P* value^4^	
	LH	HL	SEM	LH^3^	HL^3^	SEM	Direction^5^	Direction × Sex
Nutrient disappearance,^6^ %								
ATTD DM	83.9	82.8	0.17	−3.82*	−2.43*	0.260	0.003	—
ATTD nitrogen	83.3	80.2	0.30	−9.84*	−4.93*	0.468	<0.001	—
ATTD starch	99.8	99.8	0.01	−0.14*	−0.35*	0.041	0.002	—
Total tract starch fermentation^7^	11.8	18.1	0.52	17.6*	8.18*	0.013	<0.001	—
Feeding behavior^8^								
ADFI, g/d	1734	1751	35.9	−106*	−18.9	37.2	0.014	0.001
Meal frequency, *n*/d	18.0	19.3	0.44	0.57	−0.33	0.37	0.093	0.167
Meal size, g	103.9	91.0	3.76	−12.6*	5.82	3.14	<0.001	0.063
Meal duration, min	5.22	4.94	0.14	−0.09	0.03	0.12	0.472	0.160
Intermeal interval, min	75.3	67.1	2.11	−3.91*	3.84*	1.82	0.005	0.484
Feeding time, min/d	70.2	71.5	1.64	−0.18	−0.31	1.22	0.935	0.467
Visit frequency, *n*/d	43.9	44.7	1.95	0.27	2.91	1.55	0.231	0.807
Rate of feed intake, g/min	25.1	25.3	0.55	−1.19*	−0.53	0.39	0.245	0.004

1 Data are presented as least square means ± pooled SEMs. Dietary inclusion of starch sources was 50% (w/w as fed) in all diets. ADFI, average daily feed intake; ATTD, apparent total tract digestibility; DM, dry matter; HL, high RS to low RS titration; HRS, high resistant starch diet; LH, low RS to high RS titration; LRS, low resistant starch diet; TT, total tract.

2 For both titration directions, response sizes were calculated from the model ${\rm{\ }}y\ = {\rm{\ }}a + {\rm{\ }}\beta x$, where *y* = mean of response variable, *a* = intercept (0% inclusion of HRS), $\beta $ = response per percentage inclusion of HRS (*x*). Response sizes are presented as the change in each response variable corresponding to the full substitution of LRS with HRS (0% HRS to 100% HRS). Responses are corrected for the time-related effect on parameter measured in control groups (nutrient disappearance: *n* = 6; feeding behavior: *n* = 69) receiving a diet of 50% HRS and 50% LRS for the complete duration of the experiment.

3 Asterisk indicates *P *< 0.05 (≠0).

4 No significant effect of body weight class was observed.

5 Model established *P* values for fixed effects of titration direction (LH or HL).

6 *n* = 9 replicates (6 pigs/replicate).

7 Calculated from the contrast in natural ^13^C enrichment between starch and nonstarch dietary components, by assuming 5 g starch fermented/g C from starch fermentation in feces (29).

8 *n* = 105 pigs.

### Feeding behavior

#### Changes over time (control group)

Average daily feed intake of control animals increased by 15.1 g/d (*P *< 0.001), whereas meal frequency decreased by 0.09 meal/d (*P *= 0.001) (Supplemental Table 1). This resulted in an increase of 1.72 g in meal size (*P *< 0.001). Per day, meal duration increased by 0.02 min (*P* < 0.001), intermeal interval by 0.49 min (*P* = 0.005), and rate of feed intake by 0.27 g/min (*P* < 0.001).

#### Titration direction

**FIGURE 2:**
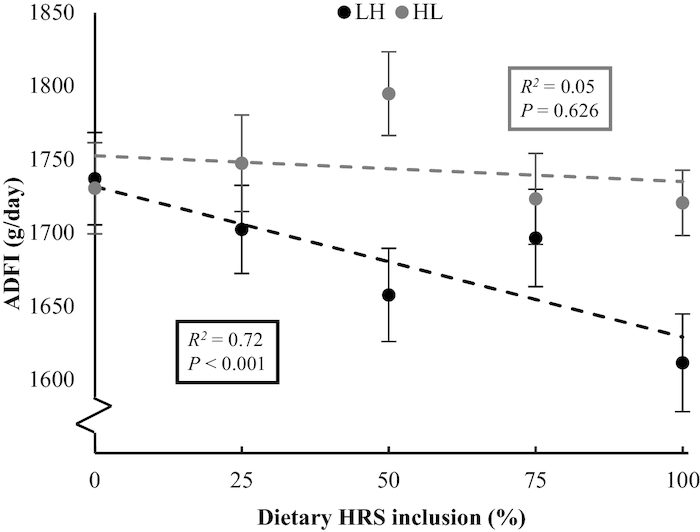
Response in ADFI of growing pigs to incremental dietary concentrations of high amylose maize starch (HRS), when substituting waxy maize starch (LRS) with high amylose maize starch (low to high RS; LH) or vice versa (high to low RS; HL) in 5 steps from 0% to 100% over a period of 28 d. Dietary inclusion of starch sources was 50% (w/w as fed) in all diets. Data were corrected for the time-related effect on parameters measured in a control group (*n* = 69 pigs) receiving a diet of 50% HRS and 50% LRS during the complete experiment. Data are presented as least square means ± pooled SEM, *n* = 105 pigs. ADFI, average daily feed intake; HL, high RS to low RS titration; HRS, high resistant starch; LH, low RS to high RS titration; LRS, low resistant starch.

For LH pigs, the complete substitution of LRS with HRS decreased ADFI, RFI, meal size, and intermeal interval (*P *< 0.050) (Table 2; **Figure 2**). The decrease in both ADFI and RFI was greater in LH females than in LH males (*P *< 0.010). The decrease in meal size tended to be greater in LH females than in LH males (*P *= 0.063). In HL pigs, intermeal interval (*P *= 0.043) increased, and daily number of visits and meal size tended to increase (*P *< 0.100). Observed responses for ADFI, meal size, and intermeal interval in HL pigs were different from those in LH pigs (*P *< 0.050).

### Correlation between response parameters

**FIGURE 3:**
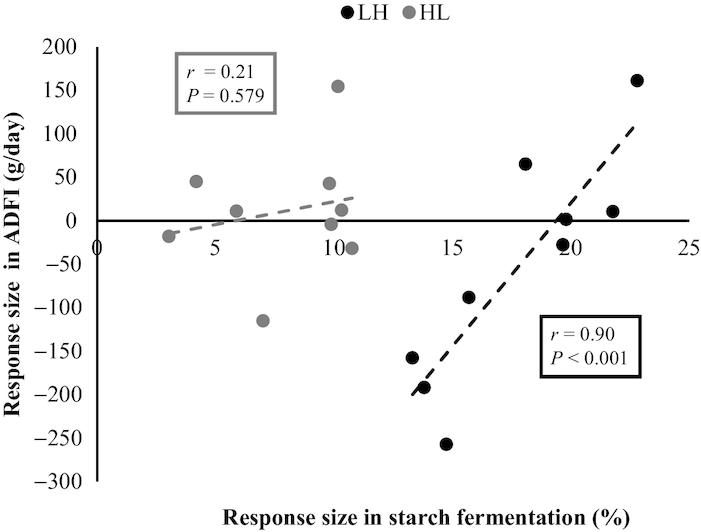
The relation between the responses in starch fermentation and ADFI to increasing dietary concentrations of high amylose maize starch (HRS) of growing pigs, which were estimated by substituting waxy maize starch (LRS) with high amylose maize starch (low to high RS; LH) or vice versa (high to low RS; HL) in 5 steps from 0% to 100% over a period of 28 d. Pearson correlation coefficients (*r*) were estimated. Dietary inclusion of starch sources was 50% (w/w as fed) in all diets fed during each step. Starch fermentation was calculated from the contrast in natural ^13^C enrichment between starch and nonstarch dietary components, by assuming 5 g starch fermented/g C from starch fermentation in feces (29); *n* = 9 replicates (6 pigs/replicate). ADFI, average daily feed intake; HL, high RS to low RS titration; HRS, high resistant starch; LH, low RS to high RS titration; LRS, low resistant starch.

In HL pigs, the response size for starch fermentation negatively correlated with the response size for ATTD of DM (*r *= −0.70, *P *= 0.035) and nitrogen (*r* = −0.81, *P *= 0.008). No such correlation was observed in LH pigs. In LH pigs, the response size for starch fermentation positively correlated with the response size for ADFI, whereas no correlation between these variables was observed in HL pigs (**Figure 3**).

## Discussion

In this study, we investigated the effects of short-term changes in dietary RS on feeding behavior of pigs, by gradually substituting LRS with HRS in a 5-step titration. Titration was executed in upward and downward directions, to study anticipated differences in the adaptation process that are required to shift from enzymatic digestion to microbial fermentation and vice versa.

### Digestive processes and feeding behavior over time

The response of the control group accounted for the anticipated increase in digestive and feeding capacity over time. The high ATTD of starch (∼100%) demonstrates the large starch fermentation capacity of the colon, resulting in very low quantities of starch being excreted in the feces. Consequently, the decrease in starch fermentation over time demonstrates the adaptation of enzymatic starch digestion, leaving less starch to be fermented. This indicates that starch digestion capacity still increases in 10-wk-old pigs, which is in line with the increase in carbohydrase activity that can be observed in pigs ≤200 d of age (35). Like starch, the ATTD of nitrogen increased over time, which may indicate that the enzymatic digestion capacity of nitrogen too is still increasing after 10 wk of age. Alternatively, the increase in the ATTD of nitrogen may result from the decrease in starch fermentation. Microbial fermentation increases fecal nitrogen excretion by stimulating microbial biomass formation (36), and ultimately increasing the influx of urea into the large intestine (37, 38). The increase in feed intake, meal size, meal duration, and RFI with time, and the decrease in meal frequency with time, are in line with earlier findings (39, 40), and are presumably explained by the greater body weight, and thus, feeding capacity, as the animals grew.

### Adaptation of digestive processes to RS intake

RS is used as a substrate for microbial fermentation (29, 36, 41), increasing starch fermentation when LRS was substituted with HRS. Increased microbial biomass (36) and urea influx (37, 38), resulting from fermentation of RS, consequently decreased the ATTD of DM and nitrogen. The responses for these parameters, however, were smaller in HL pigs than in LH pigs, indicating that the adaptation of the processes required to switch from starch fermentation to enzymatic starch digestion takes more time than vice versa. As a result, starch fermentation in HL pigs at the final titration step with 0% HRS was ∼40% greater than the initial starch fermentation at 0% HRS in LH pigs. Starch disappearance was, however, similar for both titration directions, which suggests that potentially digestible starch was fermented by HL pigs. It is possible that the increased microbial activity stimulated by high RS diets fed during the first titration steps resulted in excessive fermentation of starch during subsequent steps when low RS diets were fed. To our knowledge, this phenomenon has not yet been addressed in the literature; studies into adaptation processes in pigs after dietary changes in fermentable substrate have only considered increasing dietary concentrations of such substrates but not decreasing concentrations [e.g., (42–44)]. In contrast, starch fermentation in HL pigs that received a diet with 100% HRS at the initial titration step was ∼15% smaller than starch fermentation in LH pigs at the final titration step (Figure 1). This may indicate that an 8-d adaptation before the start of the titration was insufficient for HL pigs to fully adapt to the 100% HRS diet. The time required for full adaptation to RS is difficult to establish, and the length of the period in which pigs are allowed to adapt to diets containing RS varies among studies (from 5 to 21 d) (10, 29, 36, 45). Our results, however, show that the time required to adapt to a diet that contains RS may vary, as the rate of adaptation depends on the RS concentration in the previous diet. RS-stimulated microbiota in the small intestine still present after RS reduction probably compete with host enzymes for potentially digestible starch when switching from HRS to LRS.

### RS intake and feeding behavior

Changes in most feed intake parameters with increasing dietary concentrations of HRS were minor (e.g., RFI and intermeal interval); however, ADFI decreased (6%) for LH pigs, primarily in females, which coincided with a decrease in meal size (12%). In contrast, the ADFI of HL pigs did not change, consistent with a smaller increase in starch fermentation than in LH pigs. In studies by Da Silva et al. (19) and Doti et al. (46) daily feed intake was unaffected by dietary RS concentration (18–36%) in growing pigs (30–110 kg), whereas meal size increased and meal frequency reduced (19). In contrast, ingestion of RS reduced short-term food intake by 6.5% in humans (47), which was assessed by providing a test meal ad libitum in the evening after a fixed preload of RS during breakfast and lunch (48). The difference in findings among studies may be related to the duration of RS intake, and thus to variation in the degree of adaptation to a greater dietary RS concentration. Also, the variation in extent and site of starch digestion of the various sources used may have differently affected feeding behavior (49). The greater decrease in ADFI and meal size in female LH pigs than in male LH pigs indicates that female pigs are less capable of maintaining their feed intake when the dietary RS concentration increases; however, no evidence in pigs is available that supports this observation. In humans, however, short-chain fructo-oligosaccharides that are fermented in the large intestine reduced food intake in women, whereas in men, daily food intake was increased (50). This may be related to a slower gastric emptying and small intestinal transit of digesta in women than in men (51–53), enhancing the short-term satiating effect of RS, particularly in women.

### Relation between the degree of starch fermentation and feeding behavior

In LH pigs, the response size of ADFI was positively correlated with the response size for starch fermentation. This suggests that LH pigs that could not immediately increase starch fermentation following an increase in RS intake reduced their feed intake, whereas LH pigs that adapted more adequately were able to maintain or even increase their feed intake (Figure 3). These results indicate that the adequacy of adaptation, rather than the increase in dietary RS intake itself, explains the decrease in feed intake and meal size in LH pigs. The response size in total tract starch digestion in LH pigs, however, did not correlate with the response size in starch fermentation. This suggests that potentially digestible starch is fermented by LH pigs that quickly adapt to the increase in dietary RS, either because of an increased flow of digestible starch from the small intestine into the large intestine or by increased microbial activity in the small intestine.

### Conclusions

Increasing dietary RS reduced enzymatic starch digestion and increased starch fermentation by 18%-units, when LRS was completely substituted with HRS. A reverse substitution of LRS with HRS, however, attenuated these responses, indicating that pigs adapt slower to dietary supply of digestible starch than to supply of fermentable starch. Consequently, part of the starch that appeared to be enzymatically digestible when pigs received incremental amounts of RS was fermented when pigs were switched from HRS to LRS diets. These findings suggest that small intestinal fermentation may be more important than currently assumed and that the rate of adaptation to dietary changes in RS depends on diet history. In addition, feed intake and feeding behavior only changed in pigs poorly adapting to RS, indicating that adequacy of adaptation to RS, rather than RS itself, is an important driver for feed intake, and that feed intake responses to dietary RS supplementation seem rather transient. Our findings stress the importance of diet history for nutrient digestion and fermentation, and feeding behavior, and thus may have serious implications for nutrition research and advice.

## Supplementary Material

nxz072_Supplemental_File

## Notes

Supported by Wageningen University, Wageningen, The Netherlands, and Trouw Nutrition, Amersfoort, The Netherlands.

Author disclosures: RJJvE and TATGvK are employees of Trouw Nutrition; SdV and WJJG, no conflicts of interest.

Supplemental Figures 1–3 and Supplemental Table 1 are available from the “Supplementary data” link in the online posting of the article and from the same link in the online table of contents at https://academic.oup.com/jn/.

Abbreviations used: ADFI, average daily feed intake; ATTD, apparent total tract digestibility; DM, dry matter; EFS, electronic feeding station; HL, high resistant starch to low resistant starch titration; HRS, high resistant starch diet; LH, low resistant starch to high resistant starch titration; LRS, low resistant starch diet; RFI, rate of feed intake; RS, resistant starch.
