# Supplementary material for: Pigs Ferment Enzymatically Digestible Starch when it Is Substituted for Resistant Starch
Source: J Nutr. 2019 Jun 4;149(8):1346–53. doi: 10.1093/jn/nxz072 (PMC6686059; doi:10.1093/jn/nxz072)
Supplement: nxz072_Supplemental_File [file nxz072_supplemental_file.pdf]

Supplementary data

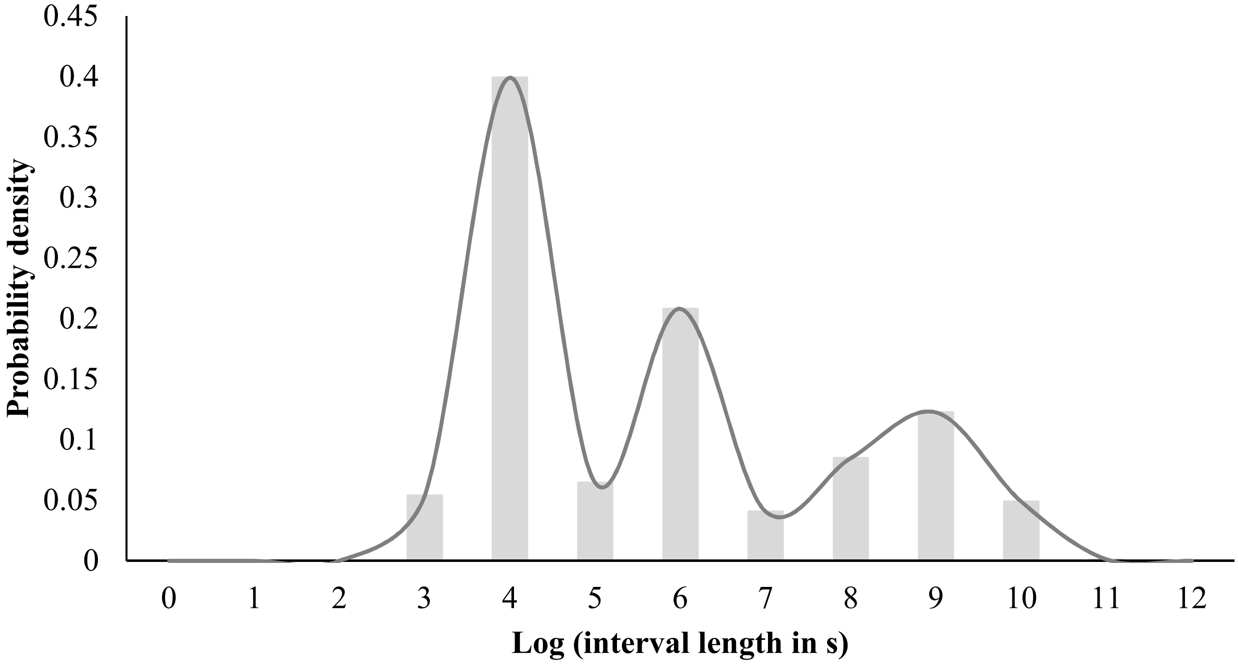

Supplemental figure 1. Typical probability density function of the model:
$y = p\left(1/\sigma_1\sqrt{2\pi}\,\mathrm{e}^{-(x-\mu_1)^2/2\sigma_1{}^2}\right) + q\left(1/\sigma_1\sqrt{2\pi}\,\mathrm{e}^{-(x-\mu_2)^2/2\sigma_2{}^2}\right) + (1-p-q)\left(\propto/\beta^{\propto}\right)x^{\propto-1}e^{-(x/\beta)^{\propto}}$,
where y is the probability density of log (interval length) in seconds, p, q, and 1-p-q are the proportions of intervals in each distribution, χ is the log (interval length) in seconds, σ_1 and σ_2, and μ_1 and μ_2, are the respective standard deviation and mean of the first and second distribution, and α and β are the respective scale and shape parameter of the third distribution.

Supplementary data

Supplemental table 1. Responses in nutrient disappearance and feed intake behaviour per day in growing pigs fed a diet containing 50% waxy maize starch and 50% high amylose maize during a period of 28 days (control group). Data is presented as least square means ± SEM[1,2]

	Intercept (day 0)			Response (Per day)[6]			*P*-value[7,8]
							BW-class
Nutrient disappearance (%)[3]							
ATTD DM	82.1	±	0.14	0.08*	±	0.007	0.001
ATTD Nitrogen	78.0	±	0.68	0.19*	±	0.013	0.001
ATTD Starch	99.7	±	0.01	0.01*	±	0.001	0.011
TT Starch fermentation[5]	21.7	±	0.81	-0.12*	±	0.031	0.029
Feed intake behaviour[4]							
ADFI (g/d)	1700	±	28.5	15.1*	±	1.41	0.410
Meal frequency (n/d)	18.0	±	0.51	-0.09*	±	0.02	0.307
Meal size (g)	102	±	3.91	1.72*	±	0.18	0.078
Meal duration (min)	5.37	±	0.17	0.02*	±	0.00	0.298
Inter-meal interval (min)	76.4	±	2.76	0.49*	±	0.13	0.161
Feeding time (min/d)	72.6	±	1.24	-0.10	±	0.05	0.818
Visit frequency (n/d)	44.0	±	1.89	-0.05	±	0.06	0.029
Rate of feed intake (g/min)	24.3	±	0.53	0.27*	±	0.02	0.504

[1] ATTD, apparent total tract digestibility; ADFI, average daily feed intake; TT, total tract;
[2] Dietary inclusion of starch sources was 50% (w/w as fed);
[3] n=6 replicates (6 pigs/replicate);
[4] n=69 pigs;
[5] Calculated from the contrast in natural ^{13}C-enrichment between starch and non-starch dietary components, by assuming 5 g starch fermented/g C from starch fermentation in feces [29];
[6] Asterisk indicates $P<0.05$ ($\mu \neq 0$);
[7] Model established p-values for fixed effects of body weight class;
[8] No significant effect of sex was observed.

Supplementary data

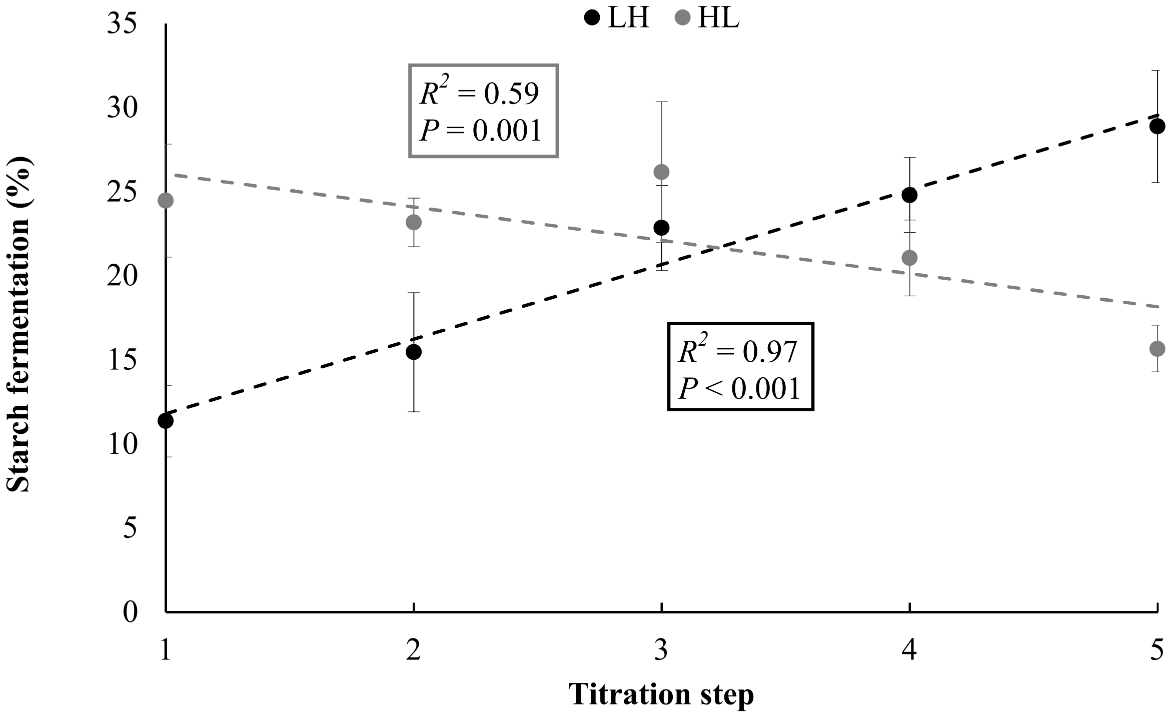

Supplemental figure 2. Response in dietary starch fermentation in growing pigs per titration step, when substituting waxy maize starch (LRS) with high amylose maize starch (low to high RS; LH) or vice versa (high to low RS; HL) in 5 steps from 0-100% over a period of 28 days. Dietary inclusion of starch sources was 50% (w/w, as fed) in all diets. Starch fermentation was calculated from the contrast in natural ^{13}C-enrichment between starch and non-starch dietary components, by assuming 5 g starch fermented/g C from starch fermentation in feces [29]. Data were corrected for the time-related effect on starch fermentation measured in control groups (n=6) receiving a diet of 50% waxy maize starch and 50% high amylose maize starch during the complete experiment. Data are presented as least square means ± SEM, n=9 replicates (group of 6 pigs).

Supplementary data

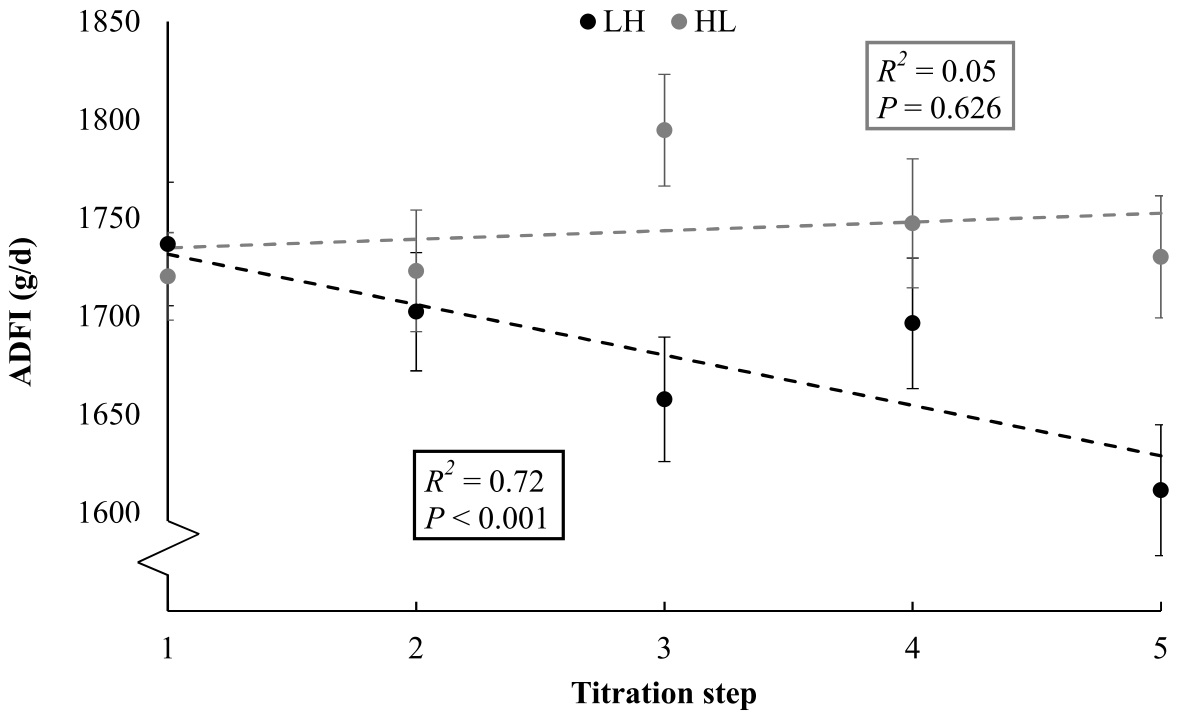

Supplemental figure 3. Response in average daily feed intake (ADFI) of growing pigs per titration step, when substituting waxy maize starch with high amylose maize starch (low to high RS; LH) or vice versa (high to low RS; HL) in 5 steps from 0-100% over a period of 28 days. Dietary inclusion of starch sources was 50% (w/w as fed) in all diets. Data were corrected for the time-related effect on parameters measured in a control groups (n=69 pigs) receiving a diet of 50% waxy maize starch and 50% high amylose maize starch during the complete experiment. Data are presented as least square means ± SEM, n=105 pigs.
